# The Impact of Uterine Radiation on Subsequent Fertility and Pregnancy Outcomes

**DOI:** 10.1155/2014/482968

**Published:** 2014-08-06

**Authors:** Wan Tinn Teh, Catharyn Stern, Sarat Chander, Martha Hickey

**Affiliations:** ^1^Department of Obstetrics & Gynaecology, University of Melbourne and the Royal Women's Hospital, Parkville, VIC 3052, Australia; ^2^Reproductive Services, Royal Women's Hospital, Parkville, VIC 3052, Australia; ^3^Melbourne IVF, East Melbourne, VIC 3002, Australia; ^4^Division of Radiation Oncology and Cancer Imaging, Peter MacCallum Cancer Centre, East Melbourne, VIC 3002, Australia

## Abstract

Future fertility is of paramount importance to younger cancer survivors. Advances in assisted reproductive technology mean that young women treated with radiation involving the uterus may require clinical guidance regarding whether to attempt a pregnancy themselves. We performed a review of the literature regarding radiation involving uterus (total body irradiation (TBI) and pelvic radiation), fertility, and pregnancy outcomes to come up with a recommendation for our patients. Limited evidence suggests lower fecundity and an increased incidence of pregnancy complications after uterine radiation. Higher radiation doses and direct uterine radiation both significantly increase the risk of an adverse pregnancy outcome. Uterine radiation doses of <4 Gy do not appear to impair uterine function. Adult TBI data (usually 12 Gy) suggest pregnancy is possible but with lower fecundity and more complications. Although there is no clear data indicating the dose of radiation to the uterus, above which a pregnancy would not be sustainable, we suggest patients receiving >45 Gy during adulthood and >25 Gy in childhood be counselled to avoid attempting pregnancy. There is preliminary evidence that menopausal hormone therapy and a combination of pentoxifylline and tocopherol may improve uterine function following irradiation.

## 1. Introduction

Cancer is a leading cause of death in developed countries [1]. Treatment of cancer, in particular cancers of the pelvic or abdominal organs, may include radiation to the uterus by one of several means. The uterus may be included, partially or whole, in the Clinical Target Volume and hence lie within the high dose region. It may be inadvertently exposed to low dose radiation by the “exit beam” from conformal radiation, with total body radiation (TBI) or in intensity modulated radiotherapy. In women, most cancers occur in the postreproductive phase, but a significant minority are diagnosed in childhood and in adult women of reproductive age. Up to 50% of cervical, 10% of anal, 5% of colorectal, 2% of uterine or bladder cancer, and soft tissue tumours/sarcomas occur in women of reproductive age and treatment commonly includes radiation involving the uterus [1, 2]. Haematological neoplasm is also not an uncommon occurrence in young girls and women. TBI is often offered as part of a conditioning regime before stem cell or bone marrow transplant treatment [1, 2]. This radiation commonly affects female fertility and when these malignancies occur in premenopausal women, the oncologist should discuss the likely consequences for fertility and the potential risks in pregnancy [3]. Referral to a fertility specialist may also be helpful.

The short term therapeutic effect of radiation is to induce cancer cell death. However, there are also delayed effects from radiation, including progressive radiation-induced injury characterized by scarring, atrophy, and sclerosis of blood vessels [4]. Depending on the dose and site, radiation can have a long-term impact on reproductive potential for cancer survivors. This may include direct irreversible radiation injury to the ovary and uterus causing sterility and disruption of hypothalamic-pituitary-ovarian axis function from cranial radiation [5, 6].

As cancer treatments improve, optimising quality of life in cancer survivors is of growing significance. Loss of fertility is a key issue for younger cancer survivors [7, 8]. Irradiation of the ovaries will induce ovarian failure in almost 90% of women, as observed in childhood abdominal radiation [5]. Ovarian transposition, shielding, or transplantation can be offered to try and reduce the risk of radiation-induced ovarian damage but the efficacy of this intervention is variable and not well established [9]. With the wider availability of assisted reproductive technology, there are commonly opportunities for ovarian stimulation and oocyte or embryo cryopreservation prior to chemoradiation. However, for those women who subsequently wish to utilise stored oocytes or embryos to achieve a pregnancy, there is very little evidence to guide whether the irradiated uterus can successfully and safely carry a pregnancy or whether surrogacy should be advised. Future options for fertility preservation after uterine radiation might potentially include uterine transplantation or cryopreservation, but these are not currently available in clinical practice. Collection of data regarding the effects of radiation on the reproductive potential of the uterus (i.e., both the endometrium and the myometrium) will assist clinicians with making appropriate clinical judgements and treatment recommendations and this is currently underway at our centre.

## 2. Physiological Effects of Uterine Irradiation

An adult size uterus measures about 7.5 cm in length, 5 cm in breadth at its upper part, and about 2.5 cm in thickness; it weighs from 30 to 40 gm [10]. During puberty, the growth of the uterus commences before the appearance of external sexual characteristics. Uterine volume increases throughout pubertal progression, with the greatest increase occurring between Tanner stage 3 and 4. Data from normal populations are limited, but uterine growth may not be completed until around 7 years after menarche at the age of 20 years [11, 12]. There is a significant increase in the uterine artery flow velocity during puberty, with measurable diastolic flow found in 35% of prepubertal girls and 100% of adult women [13].

Histological examination of the directly irradiated uterus demonstrates atrophic myometrium, with fibrosis most prominent in the inner (submucosal) half and oedema at the serosal surface. The irradiated endometrium is atrophic, with thickened and smaller blood vessels [14]. Hence, radiation may reduce reproductive potential by damaging the myometrium, the endometrium, and the uterine vasculature. The uterine volume in women with premature ovarian failure (POF) is often reduced, with poor blood flow and a thin endometrium [15–18]. Radiation exposure may induce further damage, resulting in a reduced uterine volume and decreased elasticity of uterine musculature [16, 17]. Direct high dose radiation (>25 Gy) in children commonly leads to irreversible damage to both vasculature and muscular function of the uterus [15].

## 3. Assessing Morphological Changes of Irradiated Uteri

### 3.1. Ultrasonography

Evidence for the value of ultrasound in the assessment of the postirradiated uterus comes predominantly from studies of childhood radiation. It is not known whether these observations can be extrapolated to women who have undergone uterine irradiation in adulthood. Ultrasound has been used to measure uterine volume and endometrial thickness and to assess the uterine vasculature. Several studies have examined uterine characteristics in women who had been exposed to childhood radiation by using Doppler ultrasound.

Critchley et al. assessed uterine length and blood flow in women with ovarian failure following whole abdominal irradiation in childhood. She demonstrated that mean uterine length was significantly less (*P* < 0.01) in the radiation group (mean 4.1 cm) compared with women with adult onset premature ovarian insufficiency (POI) with no history of radiation (mean 7.3 cm), and the majority (70%) of women who had been irradiated demonstrated no detectable uterine blood flow in the uterine arteries. The spontaneous POI group continued to demonstrate normal uterine blood flow [16].

These findings are consistent with other small studies in leukaemia survivors with ovarian failure following total body irradiation (TBI) in childhood, which demonstrated that uterine volume was reduced and blood flow impaired during adult life [17, 18]. Bath et al. also demonstrated absent endometrium on ultrasound in those who had been exposed to TBI in childhood. Interestingly, this study also described a correlation between the uterine size and the age of women at irradiation; that is, irradiation at a young age prepubertally was associated with a smaller uterus in adulthood [17]. It is however unclear in these women whether the reduced uterine volume is a result of direct radiation damage, reflects hormonal depletion due to associated ovarian failure, or a combination of both.

A more recent Danish study by Larsen et al. involving a larger cohort (*n* = 100) of women examined the ultrasonic characteristics in 80 nulliparous childhood cancer survivors. This confirmed that uterine volume was reduced in women who had received radiation compared to those who had chemotherapy only but also showed that direct uterine irradiation was associated with a smaller uterine volume than indirect radiation. The great majority (5/6) of those who had direct uterine irradiation with preserved ovarian function also had significantly reduced uterine volumes. This finding suggests a direct effect of radiation on the uterine musculature and vasculature following direct uterine irradiation. Luteal endometrial thickness was not measured so it is unclear from this study how direct irradiation affected the endometrium [15].

### 3.2. Magnetic Resonance Imaging

Magnetic resonance imaging (MRI) of the female pelvis can provide morphological information with excellent tissue contrast and therefore can be used to demonstrate radiation-induced changes in the uterus. Arrive et al. studied the appearance of the irradiated uterus on magnetic resonance (MR) images in women who had undergone pelvic radiation therapy (radiation dose ranged from 40 to 65 Gy) during adulthood. Radiation changes on myometrium, demonstrated by a decrease in signal intensity of the myometrium on T2-predominant MR images, can be seen as early as 1 month after therapy. Similar to findings in other studies involving women who had childhood or adolescent radiation, exposure to radiation during adulthood reduces uterine volume, and this is demonstrable 3 months after completion of radiation therapy. Endometrial changes from radiation injury, including decrease in thickness and signal intensity of the endometrium, can also be demonstrated on MRI, 6 months after therapy. The other characteristic of radiation-induced changes detectable by MRI is the loss of uterine zonal anatomy. These changes reflect myometrial and endometrial atrophy, fibrosis, and local tissue ischaemia. These postirradiation MR changes are similar to the changes ordinarily seen on MRI of the normal postmenopausal uterus [14].

In summary, radiation-induced changes by ultrasound or MRI examination can be detected by one month after therapy completion. Current evidence from observational studies using ultrasound or MRI assessment has suggested that radiation exposure will affect the myometrium (reduce uterine volume), the endometrium (reduced endometrial thickness), and the uterine vasculature (impaired uterine blood flow), with the most significant effect seen in those who require direct uterine irradiation or radiation at a younger age.

## 4. Fertility and Pregnancy Outcomes following Uterine Irradiation

### 4.1. Childhood and Adolescent Radiation Exposure

It is well established that cancer survivors have a lower fecundity and face an increased risk of adverse outcomes in pregnancy [19–23], attributed to the associated cancer treatments, including surgery, chemotherapy, and radiation therapy. Multiple cohort studies have demonstrated an increased risk of pregnancy complications (miscarriage, preterm delivery, low birth weight, and perinatal death) in women previously exposed to childhood abdominal radiation [24–27]. Even low doses of radiation can have a negative impact on future fertility. A dose of below 4 Gy appears to be the threshold dose, depending also on the associated treatment. If the uterus is directly irradiated, pregnancy is rare. Childhood radiation doses of <4 Gy have not been shown to impact negatively on subsequent fertility [28].

### 4.2. Adulthood Radiation Exposure

The continued refinements in cancer treatments have significantly improved the cure rates of many young women with malignancies including haematological, cervical, and colorectal cancer which may include uterine irradiation. Almost all the available information about the impact of radiation on the uterus comes from radiation exposure during childhood or adolescence, and it is not known whether this data can be extrapolated to women undergoing uterine radiation in adulthood.

#### 4.2.1. Total Body Irradiation

Current evidence concerning pregnancy outcomes in women who had uterine radiation beyond childhood is largely limited to those who had TBI as a conditioning regimen before stem cell transplantation (SCT) or bone marrow transplantation (BMT). Sanders et al. followed up pregnancy outcomes in patients who had received high dose chemotherapy alone or with TBI and BMT for aplastic anaemia or hematologic malignancy. The incidence of spontaneous abortion (37% versus 7%) and preterm delivery (63% versus 18%) were significantly higher in TBI recipients when compared to the chemotherapy-only group (*P* = 0.01). The 13 preterm deliveries resulted in 10 low birth weight (1.8 to 2.24 kg) and three very low birth weight (≤1.36 kg) infants, for an overall incidence of 25%, which is higher than the expected incidence of 6.5% for the general population (*P* = 0.0001). Although the analyses involved both pre- and postpubertal women, the majority of women (87%) were postpubertal at the time of BMT. This data demonstrated that female recipients of BMT had a high incidence of miscarriage, premature labour, and LBW offspring, with the risk higher in those who also received pretransplant TBI [29]. However, it was unclear whether these pregnancies were the result of spontaneous conception or assisted reproductive technologies (ART).

In a large study of more than 30,000 European women who had received SCT, there were 312 pregnancies from 232 patients (30 patients had ART). This study demonstrated a significantly higher than normal rate of pregnancy complications in recipients of allogeneic SCT compared to the normal population. These increased pregnancy risks were confined to those who had received total body irradiation in their pretransplant conditioning and were most striking in those who had conceived via ART. Analysis of singleton pregnancies has shown that these women had significantly higher rates of caesarean section (42% versus 16%), preterm delivery (20% versus 6%), and low birth weight singleton offspring (23% versus 6%) compared to the general population [30].

Carter et al. compared the pregnancy outcomes in adult survivors of haematological malignancies to their closest age siblings. Less pregnancy was reported in survivors than their siblings (3% in female survivors, 72% in female siblings, *P* < 0.0001). Those who had SCT at older age or had exposure to TBI were more likely to be nulliparous. Interestingly, this study found no significant increased prevalence of pregnancy complications including miscarriage, preterm birth, and stillbirths. However, the number of participants in this study is smaller with only 14 reported pregnancies in 8 female survivors, when compared to the previous studies. This study did not include information regarding assisted reproductive technologies which might have been utilised by survivors to achieve pregnancy [31].

#### 4.2.2. Pelvic Radiation

There is minimal data in the literature about fertility and pregnancy outcome of women who had adulthood pelvic radiation. Bath et al. reported a case of successful pregnancy in a 25- year-old women who received pelvic chemoradiotherapy (30 Gy) for anal cancer [32]. Another pregnancy was reported in a woman who received radiation (36 Gy in 18 fractions) to the right hemipelvis for Hodgkin's disease at age 16. The patient conceived a dichorionic twin pregnancy via a donor oocyte program 15 years later. Her pregnancy was complicated by preeclampsia and preterm delivery at 35 weeks of gestation. At caesarean section, the right lateral placenta was morbidly adherent to the endometrium. It was suggested that the morbidly adherent placenta could be the result of endometrial damage from the previous right hemipelvic radiation [33]. On the other hand, a successful spontaneous pregnancy was reported in a woman who was exposed to pelvic radiation (55 Gy to left semipelvis and 10 Gy to right semipelvis) at the age of 14 for Ewing sarcoma [34].

### 4.3. Assisted Reproductive Technology

Vernaeve et al. reported fertility and pregnancy outcome of a cohort of 15 women with a past history of postpubertal pelvic radiation (8) or TBI (7) in an oocyte donor program. These recipients were given oral oestradiol in a progressively escalating dose regimen for the endometrial preparation. However, endometrial thickness was not routinely measured before embryo transfer. Out of the 15 patients, 8 became pregnant (53.3%) and 7 had an ongoing pregnancy at the time of the study. An implantation rate of 31% was observed among these women, which was comparable to the unit's general oocyte recipient implantation rate. However, a higher rate of pregnancy complications (miscarriage, preeclampsia, premature delivery, and placental haemorrhage and stillbirth) was observed in this subgroup of oocyte recipients [34].

Within the last 20 years, more than 100 women have been referred for consultation at the fertility preservation service at the Royal Women's Hospital and Melbourne IVF, prior to pelvic radiation (89 had TBI, 16 had pelvic radiation). However, not all of these women wanted to conceive. 33% of women in the TBI group versus 19% of those who had pelvic radiation reported spontaneous menstruation after their treatments. Five live births have been reported in women who had TBI (age at diagnosis 7–39 (mean 27) year old). There are no reports of pregnancies following pelvic radiation (age at diagnosis 21–40 (mean 29) year old). Only 1 out of the 5 live births in women who had TBI was the result of ART. However, these are pregnancies reported by patients, and it does not represent the actual pregnancy rate in our uterine radiation population.

Although limited, the current evidence suggests that women who wish to have children and who have been exposed to radiation (TBI) are less likely to conceive and are at increased risk of pregnancy complications including preterm birth and low birth weight offspring. The increase in pregnancy complications seem to further increase when the conception results from ART. Unfortunately, there is no information in the literature about fertility and pregnancy outcome in women who have been exposed to pelvic radiation in adulthood.

## 5. Potential Modalities to Improve Uterine Function after Radiation

### 5.1. Sex Steroid Replacement

Sex steroid replacement has been given to women who have been exposed to childhood and adolescent radiation in an attempt to improve the uterine function after radiation [15–17]. Physiological sex steroid regimen in the form of oestradiol patches (100–150 *μ*g/24 h) and progesterone pessaries (400 mg/24 hr) has a greater beneficial effect upon endometrial thickness in women with POI in comparison with standard regimen with oral contraceptive pill [35]. In order to achieve uterine growth, high dose of sex steroid replacement (as above) is required. The dosage of sex steroid replacement therapy that was sufficient to induce puberty and menstruation and control menopausal symptoms was inadequate to encourage uterine growth following childhood irradiation [18].

Replacement therapy of 3 months or more increases uterine volume, increases midluteal endometrial thickness, and restores uterine vascular supply in women exposed to TBI in childhood or adolescence. Although improvements of uterine characteristics have been observed on ultrasound at 3 months of replacement therapy, the uterine volume of these women remains significantly smaller compared with controls [17]. It has been suggested that ongoing sex steroid replacement may lead to further improvements in these uterine parameters. However, a longer-term study involving 12 months of sex steroid replacement therapy in POI women has demonstrated that treatment response is generally apparent after 3 months [35]. It is also not known if improvement of these uterine parameters correlates with improved fertility and pregnancy outcome.

In women who were exposed to higher dose radiation at a young age (2–11 years; radiation dose of 25–30 Gy and 30–54 Gy in study reported by Larsen et al. and Critchley et al. resp.), no demonstrable improvement in uterine characteristics was seen with up to 3 months of sex steroid replacement therapy [15, 16]. This absence of improvement in uterine characteristics was observed despite adequate replacement therapy with physiological range of serum oestradiol and progesterone [16].

### 5.2. Pentoxifylline and Tocopherol

Radiation-induced fibrosis is mainly characterized by nonspecific changes in connective tissue, with excessive extracellular matrix deposition, excessive myofibroblast proliferation, and the presence of an inflammatory infiltrate. Recent study of the physiopathology of the radiation-induced fibroatrophic process has led to the idea of potential intervention via modulation of the antioxidant pathway. Free radicals like reactive oxygen (ROS) or nitrogen species (RNS) perform useful functions such as cell differentiation and proliferation under physiological conditions. However, excess ROS/RNS production induced by environmental factors including radiation results in pathological stress to tissue and cells that encourages fibrogenesis [36].

The combination of pentoxifylline (PTX) and tocopherol (vitamin E) has been recently shown, in both animal and human studies to induce regression of radiation-induced superficial and musculocutaneous fibrosis [37, 38]. Pentoxifylline is a methylxanthine derivative used to treat vascular diseases. In vivo, it increases erythrocyte flexibility, vasodilates, and inhibits inflammatory reactions and tumour necrosis factor. In vitro, PTX inhibits human dermal fibroblast proliferation and extracellular matrix production and increases collagenase activity [38]. The function of vitamin E is to scavenge the free radicals like reactive oxygen generated during oxidative stress and therefore reduce free radical-induced chromosomal damage [39, 40].

To test the theoretical benefit of pentoxifylline and tocopherol, Letur-Könirsch et al. conducted a phase II trial involving 6 women aged 28 to 37 years with chronic uterine radiation-induced damage. All subjects had received a total dose of 28 to 65 Gy radiation for childhood abdominopelvic tumours. Amenorrhoeic women (4 out of 6 women) were given hormone replacement in the form of transdermal oestradiol (Estraderm 100 TTS changed twice per week) and progesterone (Utrogestan 300 mg per day from day 15 to 28) for 3 months before the PTX-Vit E treatment. A booster cycle comprised of higher dose of oestradiol (two patches of Estradem 100, changed daily), together with the same regimen of progesterone, was introduced just before commencement of the PTX-Vit E treatment. Each patient was given twice daily combinations of 400 mg of pentoxifylline and 500 IU of *α*-tocopherol for at least 9 months. After 3 months of PTX-Vit E treatment, hormone replacement was reintroduced. Improvements in endometrial thickness, (6.2 ± 0.6 versus 3.2 ± 1.1 mm), myometrial dimensions (44 [±5] × 30 [±3] × 20 [±2] versus 30 [±7] × 22 [±3] × 16 [±2] mm), and diastolic uterine artery flow were observed [41].

There are case reports of pregnancies in patients following chemoradiation-induced ovarian failure, who did not respond to hormone replacement therapy, when given the combined pentoxifylline and tocopherol treatment [42–44]. 18 oocyte recipients who failed to develop a preovulatory endometrial thickness of >6 mm after receiving vaginal micronized oestradiol were given 6 months of PTX-Vit E treatment. Two of these patients had previous radiotherapy in the form of TBI (20 Gy). Five patients became pregnant. Three patients, of whom two had previously received TBI, became spontaneously pregnant during the treatment, and the other two pregnancies were from embryo transfer. Four patients had normal pregnancies and gave birth to healthy babies. One of the pregnancies from embryo transfer had a fetal death at 20 weeks of gestation due to fetal hygroma. Endometrial thickness increased significantly (*P* < 0.001), with a mean of 4.9 ± 0.6 mm before and 6.2 ± 1.4 mm after treatment. This increase in endometrial thickness was especially noticeable in patients who had previously received total body irradiation [43]. Although with small numbers, these trials have demonstrated that PTX-Vit E may play a role with improving the function of radiation-damaged uteri. The pentoxifylline and tocopherol regimen seems to be well tolerated [40]. Adverse effects that commonly occur with pentoxifylline when used for intermittent claudication include dose-related gastrointestinal (nausea, dyspepsia) and central nervous system (jitteriness, insomnia, vertigo, asthenia) effects [43]. Overall, PTX-Vit E combination is one of the few available drugs with clinical data for management of radiation-induced fibrosis. Further trials are warranted on the role of PTX-Vit E in reducing radiation-induced uterine injury due to ease of administration, favourable safety profile, and limited treatment options. Large randomized controlled trials are needed to substantiate these preliminary findings.

## 6. Conclusions and Recommendations

- Oocyte donation and fertility preservation with oocyte or embryo cryopreservation prior to cancer treatment provides significant hope for cancer survivors who undergo chemotherapy or radiation-induced ovarian failure. Appropriate counselling in regards to the safety of the irradiated uterus with carrying a pregnancy should be provided to these women who subsequently wish to utilise their stored oocytes and embryos to achieve a pregnancy. A successful pregnancy will require not only a viable embryo, but also a uterine cavity that is receptive to embryo implantation, and a uterus that has the ability to accommodate normal growth of the fetus to term.
- Previous uterine irradiation is associated with a smaller uterine volume; this can be related to direct radiation damage and/or hormonal depletion due to associated ovarian failure [15, 17].
- The threshold radiation dose for uterine damage to occur such that pregnancy is not sustainable is unknown. To our knowledge no successful pregnancy has been reported after a direct radical dose (>45 Gy) to the whole pelvis.
- It appears that younger age at uterine radiation leads to greater adverse effects on uterine reproductive capacity, particularly in prepubertal girls.
- The radiation-induced uterine injury is also dose and site dependent and with more severe uterine damage occurs with higher dose radiation and radiation directly involving the uterus. Radiation doses of >25 Gy directly to the uterus in childhood appears to induce irreversible damage [15, 16].
- Exposure of adult uterus to TBI (12 Gy) is associated with increased risk of miscarriage, preterm labour, and low birth weight babies.
- The mechanisms of impaired uterine function following radiotherapy are not clearly defined; however, impaired uterine blood supply, defective endometrial function, and poor uterine distensibility have all been implicated. It has been suggested that the uterine damage from radiation is related to (a) damage to the endometrium, therefore impairing normal decidualization and interference with placentation. (b) Damage to the uterine vasculature and impairment of future trophoblast invasion, which ultimately can decrease fetal-placental blood flow causing fetal growth restriction. (c) Development of myometrial fibrosis which reduces uterine elasticity and volume. This can lead to preterm labour and delivery [45, 46].
- There is limited evidence to guide management of women with cryopreserved oocytes or embryos following pelvic radiation. Ultrasound and Doppler examinations can be used to assess uterine volume, endometrial thickness, and uterine vascular supply but there are no values to guide clinicians about when a pregnancy could safely be attempted. MRI is an effective tool to demonstrate other characteristics of radiation-induced changes, including atrophy, fibrosis, and local tissue ischemia, but there are no published studies of uterine MRI changes before and after radiation.
- There is currently no information to show that changes in uterine imaging predict pregnancy outcomes following uterine radiation.
- Sex steroid replacement may have a role in restoration of satisfactory uterine volume, endometrial thickness, and uterine vascularization in women who have been exposed to lower dose uterine radiation (<25 Gy) at a postpubertal age. However, its use in women who had been exposed to higher dose radiation (>25 Gy) at a younger age has been disappointing [15, 16].

### 6.1. Future Potential Mechanisms to Improve/Protect Uterine Function after Radiation

**Figure 1:**
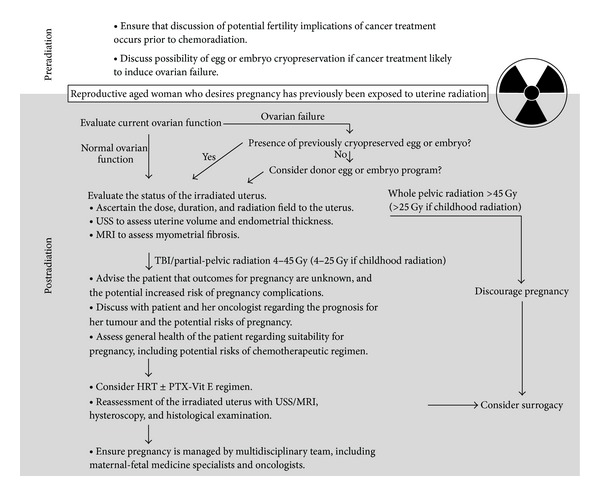
Proposed process for assessment of uterus after radiation therapy in patient requesting fertility.

- There is preliminary evidence that a combination of pentoxifylline and tocopherol may improve uterine function as an adjuvant therapy. Although successful pregnancies have been reported, more evidence is needed to demonstrate the efficacy and safety of PTX-Vit E in improving pregnancy rates and outcome in women who are suffering from radiation-induced uterine injury.
- With improvements in radiotherapy delivery technology methods such as intensity modulated radiation therapy, cyberknife, tomotherapy, and stereotactic radiotherapy, there may be the potential to limit radiotherapy exposure to the uterus or to restrict exposure to part of the uterine corpus or cervix, depending on the tumour location and characteristics. The reproductive implications of partial uterine irradiation are not yet known.
- With the growing numbers of adult women surviving cancers managed with pelvic radiation, there is an urgent need to ensure that fertility issues are discussed prior to treatment and that further data are collected on the reproductive impact of total or partial uterine radiation in order to inform future clinical management (Figure 1).
